# Fine Particulate Matter Air Pollution and Lung Function Among U.S. Older Adults

**DOI:** 10.1093/geroni/igaf122.813

**Published:** 2025-12-31

**Authors:** Sung Eun Cho, Eun Young Choi, Jennifer Ailshire

**Affiliations:** University of Southern California, Los Angeles, California, United States; University of Southern California, Los Angeles, California, United States; University of Southern California, Los Angeles, California, United States

## Abstract

Although prior research has found associations between fine particulate matter air pollution (PM2.5) and lung function among older adults, the findings have come from region-specific studies and have focused on individuals with pre-existing conditions. Furthermore, it remains unclear how individual health behaviors contribute to variations in vulnerability to PM2.5 exposure. This study examines the association between outdoor PM2.5 levels and lung function in a nationally representative and diverse sample of U.S. older adults from the 2016 and 2018 Health and Retirement Study (HRS). Our study sample includes 7,119 adults aged 65 and older linked with 2015 and 2017 census tract-level annual average PM2.5 concentrations from the HRS Contextual Data Resource. Regression models examined the cross-sectional association between PM2.5 concentration quartiles and lung function, measured through the peak flow meter tests. Older adults living in areas with the second-highest PM2.5 quartile had significantly lower lung function (b = -1.95, p = 0.013) compared to those in the lowest quartile, even after adjusting for demographic, socioeconomic, and health characteristics. Living in the highest PM2.5 quartile was not associated with lower lung function (b = -0.53, p = 0.505). However, in this quartile, those who were physically active exhibited worse lung function compared to those who were physically inactive (predicted lung function: 80.91 vs. 83.49, p = 0.000). These findings highlight the negative impact of PM2.5 on older adults’ lung health and suggest that physical activity, potentially indicative of increased outdoor exposure, may heighten vulnerability to air pollution in highly polluted areas.

